# Determination of Physicochemical Parameters and Levels of Heavy Metals in Food Waste Water with Environmental Effects

**DOI:** 10.1155/2020/8886093

**Published:** 2020-08-20

**Authors:** Jingxi Ma, Shuqing Wu, N. V. Ravi Shekhar, Supriya Biswas, Anoop Kumar Sahu

**Affiliations:** ^1^College of Foods and Technology, Changchun University, Chang'Chun 130012, China; ^2^Department of Chemistry, Kalinga University, Raipur (C.G), India; ^3^Department of Chemistry, Shri Shankaracharya Technical Campus (CSVTU), Bhilai (C.G), India; ^4^Faculty of Department of Mechanical Engineering, School of Studies in Engineering and Technology Guru Ghasidas Vishwavidyalaya (A Central University), Bilaspur, Chhattisgarh, India

## Abstract

Bioinorganic chemistry is found as a sizzling field in today's era. It deals with chemistry amongst the heavy metals with natural resources, i.e., air, soil, water, plant byproducts (foods), and environmental essences. The aim of this research is to determine the concentration of heavy metals present in the food waste water sample and to study the environmental effects of metal ion concentration. To conduct the research work, the physicochemical parameters and levels of five heavy metals of food waste water samples were collected from five sampling points of renowned hotels, restaurants, canteens, and confectionaries of a state of India and assessed using the standard analytical procedure. Sampling was carried out from January 2017 up to December 2017. The physicochemical parameters were determined such as pH, temperature, turbidity, conductivity, total dissolved solids, total suspended solids, total alkalinity, biological oxygen demand, chemical oxygen demand, dissolved oxygen, total organic carbon, sulphate, nitrate, and phosphate. The heavy metal concentration was determined by using the UV-spectrophotometer, and the results were compared with the standards prescribed by the WHO, BIS, ICMR, and municipal authorities. The results obtained in the physicochemical analysis revealed that a few parameters were found beyond limits, and the metal ion concentration (iron and zinc) results were found above the permissible limits set by the CPCB (Central Pollution Control Board), ICMR, BIS, and World Health Organization (WHO), most especially, effluent from point *P*1. It was concluded that all the effluents required further treatment before releasing them into the water body or land to prevent pollution. The obtained results reveal that waste water used for irrigation and farming of nearby areas and water drained from restaurant kitchens were considerably polluted and not suitable for aquatic organisms, irrigation, and agricultural purposes.

## 1. Introduction

In the present situation across the world, the poor quality of discharged water by restaurants, hotels, and various food-selling commercial sites contains various levels of pollutants. This poor quality of discharged water is the result of poor management of discharged food waste water. These pollutants are discharged either with intent or mistakenly into the environment, which are released straight or indirectly into public sewer lines, dumping yards, and reservoirs [1]. It is probed that heavy metals such as Pb, Cu, Fe, Ni, Hg, and Zn are found in food waste water. They not only produce toxic or chronic poisoning in aquatic animals but also pose threat to the environment. Waste water used for irrigation fields contains considerable amount of potentially toxic substances including dissolved salts and heavy metals like Fe^2+^, Cu^2+^, Zn^2+^, Mn^2+^, Ni^2+^, and Pb^2+^. It is seen that, in agriculture fields, waste water contaminates the soil to such an extent that it becomes toxic to flora and fauna. The crops and plants accumulate heavy metals of waste food water in their tissues at concentrations ranging above the acceptable levels, which is considered harmful to the ecosystem and aquatic organisms. Researchers proved that high rate of exploration and recharging, poor management of dumping of solid and liquid wastes into portable water, and lacking strict enforcement of the law and poor bye laws are the major causes of deterioration of ground water quality. Previous years, in many cities and towns, sewage water was sold out, and it was a good source of income to municipalities as sewage water is still considered to be the most rich in plant nutrients and organic matter. Nowadays, the situation has changed; sewage water is available free of cost to adjoin agricultural fields and enrich with macro- and micronutrients, which are required for the plant growth, and therefore, farmers prefer sewage irrigation for saving the cost of fertilizers and irrigation water. Besides nutrients, heavy metals are also present in waste water and lead to bioaccumulation of heavy metals in the cultivated crops, posing danger to aquatic life. It is well established that the ability of heavy metals binding to biomolecules is toxic to organisms. Some are known to be mutagens or carcinogens [2]. Different types of heavy metals are reported from terrestrial and marine wild life in Oman [3, 4]. The objective of this research is to determine the levels of some physicochemical parameters to determine the levels of heavy metal concentration and to study their environmental effects.

## 2. Materials and Methods

### 2.1. Description of the Study Area

An old bus stand area of Bilaspur city is a busy commercialized site situated in the Bilaspur district in Chhattisgarh state, India. Bilaspur is situated at 21.09°N 82.15°E, and the height of sea level is 264 meters. Old Bus Stand Square (chowk) is located in the heart of the city centre, main market area, where traffic flow is very high. The selection of the site is owing to a large number of hotels, restaurants, confectioneries, and food vending sites which are situated where outflow of food waste water and quality are of main concerns.

### 2.2. Sampling Area and Sampling Point

Food waste water samples were collected from January 2017 to December 2017 from kitchen drains of different renowned hotels and restaurants of Bilaspur area (Old Bus Stand Chowk) for the analysis of physicochemical parameters and heavy metal determination. Sampling points for the samples were designated as *P*1 to *P*5. Food wastewater samples were collected at the discharge point from Tagore Chowk designated as *P*5, 300 meters away from Old Bus Stand chowk (*P*4), and at 400 meters towards Agrasen Chowk (*P*3) and towards Telipara, samples are taken from two different hotels designated as (*P*2) and (*P*1), respectively. Food waste water is sampled at these points.

### 2.3. Sample Collection

The waste water samples were collected from the selected five locations (*P*1–*P*5) at the discharge pit unit or through kitchen drains of the hotels and restaurants designated from *P*1 to *P*5 in hard glass bottles (2 L), which are precleaned thoroughly with nonionic detergent, rinsed with tap water and after some time, soaked in 10% HNO_3_, and lastly with distilled water. The samples were collected differently for multiple tests. Ademoroti [5] stated that pH, temperature, colour, turbidity, and conductivity of waste water can be measured if collected samples of waste water are revealing the biodegradation characteristics. The samples were labeled cautiously and transported to the laboratory and stored in the refrigerator at about 4°C prior to analysis. The waste water samples used for DO and BOD determinations were collected directly into dark DO bottles, and some drops of manganous sulphate solution were added to fix dissolve oxygen. After collection, they were stored at room temperature.

### 2.4. Analytical Method

The analytical test of the collected water samples was performed for various parameters such as pH, temperature, turbidity, chemical oxygen demand (COD), biological oxygen demand (BOD), dissolved oxygen (DO), TOC, conductivity, total dissolved solid (TDS), total suspended solid (TSS), alkalinity, sulphate, nitrate, and phosphate. The sampling point for the collection of food waste water samples throughout the research was the kitchen drain of various restaurants. Observations and findings were continuously recorded for the assessment of parameters like pH, temperature, turbidity, conductivity, TDS, TSS, BOD, COD, DO, TOC, sulphate, nitrate, and phosphate to assess the nature of extent of pollution. The parameters such as total dissolved solid (TDS), biochemical oxygen demand, sulphate, nitrate, and phosphate were analyzed as per the standard guidelines and procedures as described in the standard methods from the guide manual and waste water analysis as per [6] and other reference sources. Determination of heavy metal concentration was performed analytically with the digital UV-spectrophotometer [7]. The collected samples of waste water can be maintained without harming samples biodegradation characteristic and are investigated in [8–11]. All the reagents used for the analysis were of analytical grade and obtained from Merck and Qualigens brand. Deepa et al. [12–14] suggested the standard path for comparing the collected sampling data for the water quality index, which was added in the research work [12, 13]. Published works were not experimentally dealt with food waste water sources. Hence, the collected data in the presented research work cannot be compared with [12–14].

## 3. Digestions of Waste Water Samples for Heavy Metal Determination

Thoroughly mixed sample was taken in an evaporating silica dish and acidified to methyl orange with conc. H_2_SO_4_, and 5 mL conc. HNO_3_ was also added. 2 mL of 30% H_2_O_2_ was added to reduce chromate (if any). Next, mixed sample was evaporated by 10 mL water and mixed with 5 mL conc. HNO_3_ in purpose to transfer to 125 conical flask. The content was not allowed to dry during digestion. 1-2 ml of concentrated HNO_3_ was further added to dissolve the remaining residue, and a few glass beads were also added to prevent bumping, and 50 mL distilled water was added. Solution was boiled to dissolve the solids and then filtered through the sintered glass crucible. The filtrate was transferred to a 100 mL volumetric flask and made up to the mark with distilled water. The resulting solution was of 3 N in H_2_SO_4_. Aliquots of the solution were used for the determination of metals. For determination of lead, 50 mL of ammonium acetate solution was added in the flask itself. Determination of heavy metals (iron, copper, nickel, lead, and zinc) in the waste water samples was done by preparing samples volumetrically and using a digital spectrophotometer as described in the manufacturer's instruction manual.

### 3.1. Determination of Physicochemical Parameters

The assessment of various physicochemical parameters, namely pH, temperature, turbidity, BOD, COD, DO, TOC, conductivity, TDS, TSS, total alkalinity, sulphate, nitrate, and phosphate and heavy metal concentrations (iron, copper, lead, nickel, and zinc) were carried out as per the method described in APHA [15] and guide manual [7]. The instruments used were in the limit of precised accuracy. The chemicals used were of AR grade. Due care was taken during sampling to avoid any possibility of contamination. Temperature and pH were measured in situ. Known buffer solutions of pH 4, pH 7, and pH 10 were prepared and used to standardize the equipment, and the pH readings of the water samples were immediately taken. All field meters and equipment were checked and calibrated according to the manufacturer's specifications and instructions [16]. Total dissolved solid was determined by subtracting the values of the suspended solids from the corresponding total solids of the samples. Total suspended solid was determined by using Whatman filter paper rinsed in double distilled water and was dried in an oven at 105 C for exactly one hour and cooled in desiccators. Its residue weight (*W*1) was determined using a digital balance. The sample of 100 mL of water was filtered through the resin paper and then evaporated at 105°C for one hour. This weight which represents *W*2 of the filter paper containing the residue was noted, and TSS was calculated using (*W*2 − *W*1) × 100 mg/L. Alkalinity values are determined by titration methods [15]. 50 ml of the water samples was taken in a clean 150 mL conical flask, and three drops of the phenolphthalein indicator were added. After that, it was titrated with 0.05 M H_2_SO_4_ until colour disappeared. To the colourless solution, three drops of the methyl orange indicator were added and titrated further until colour changed from yellow to permanent reddish or orange red, and then titre values were recorded, and alkalinity was calculated. Biochemical oxygen demand was determined using azide modification of Winkler's method. BOD bottle was prepared and incubated at 20°C for 5 days in the dark. After five days, incubated BOD bottle was poured with mixing 2 mL of orthophosphoric acid. This was shaken gently and titrated with sodium thiosulphate to the end point where there was change in colour. The titre value represents dissolve oxygen on day five. BOD was then calculated as the difference between dissolve oxygen on day one and that on day five. Determination of COD was done as per the method described in standard methods. 50 ml of the water sample was taken in a reflux flask, and 10 mL of potassium dichromate solution with 1 g mercuric sulphate was thoroughly mixed. Antibumping beads were added to control boiling of the solution. To this, 10 mL of concentrated sulphuric acid containing silver sulphate was added through the open end of the condenser carefully and mixed by swirling motion. The reflux apparatus was operated for around 1 hour and allowed to cool. The flask was removed, and its content was diluted to 150 mL with distilled water. To the resulting solution, three drops of the ferroin indicator were added. This sample was titrated with standard ferrous ammonium sulphate to an end point where blue-green colour just changed to reddish-brown. Chemical oxygen demand (COD) of the blank sample was then calculated. Dissolved oxygen was determined using azide modification of Winkler's method. 200 mL of the water sample was carefully transferred into a 300 ml BOD bottle. 1 mL of manganese sulphate solution was added followed by 1 mL of the alkaline alkali-iodide-azide reagent. The resulting mixture was titrated against 0.025 N sodium thiosulphate to the end point where there was colour change. The titre value was recorded as DO [17]. Determination of total organic carbon (TOC) was carried out according to the method described by standard procedures [15]. The dried sediment samples were finely pulverized, and 0.2 grams each was weighed into 500 ml conical flasks, and 10 mL of 0.5 M K_2_Cr_2_O_7_ was added and swirled gently. Concentrated H_2_SO_4_ (20 mL) was added with care directly into the suspension. The mixture was swirled gently and allowed to stand for about 40 minutes. 200 ml of distilled water was added followed by careful addition of 10 mL concentrated H_3_PO_4_. The mixture was allowed to cool, and three drops of the ferroin indicator were added. The contents were then titrated with 0.25 M FAS to the wine-red end point. (%) TOC of the water sample was calculated as per the following formula [5]: (%) TOC = (*V*_*b*_ − *V*_*s*_) × M × 1.38 = *R*/*W*, where *V*_*b*_ = volume of FAS for blank; *V*_*s*_ = volume of FAS for the sample; *M* = morality of FAS; and *W* = weight of the sample in grams. Sulphate was determined by the gravimetric/turbidimetric method using BaCl_2_ as precipitant. 50 mL of the sample was measured into a 250 mL beaker and diluted to 150 mL with distilled water. 1 mL of conc. HCl and four drops of the methyl orange indicator were added. To this, 10 mL of 10% barium chloride solution was added and then boiled for 5 minutes. The solution was retained overnight and then filtered using Whatman filter paper. The filter paper was rinsed with distilled water to free it from chloride ions. The filter paper was dried at 80°C in an oven by using the silica crucible, ignited at 800°C in a muffle furnace for 1 hour, cooled in a desiccator, and then weighed. Ignition, cooling, and weighing were repeated to give a constant value. Sulphate content of the water sample was then calculated. Determination of nitrate was done by the phenoldisulphonic acid method. Phosphate was determined by the colorimetric method. 2 ml aliquot of the water sample was taken in a 25 ml volumetric flask, and one drop of the phenolphthalein indicator was added followed by 2 mL of ammonium molybdate, and then, 1 mL of freshly diluted stannous chloride solution was added. These were made up to 25 mL volume with distilled water and mixed thoroughly. After 5-6 minutes and before 20 minutes, the colour intensity (absorbance) was measured at a wavelength of 660 nm in a spectrophotometer. Heavy metal concentration was determined analytically and spectrophotometrically by [18], and its results were correlated and compared with the WHO and municipal authorities' limit.

## 4. Results

**Figure 1:**
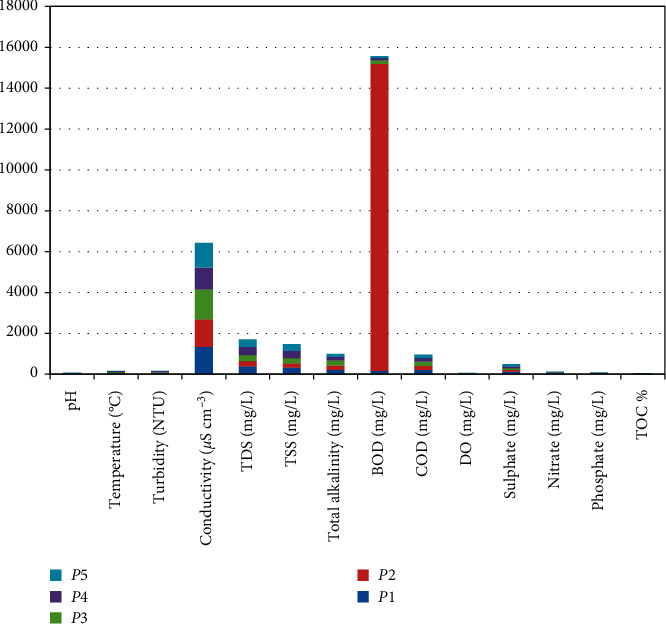
Graph of physicochemical parameters.

**Figure 2:**
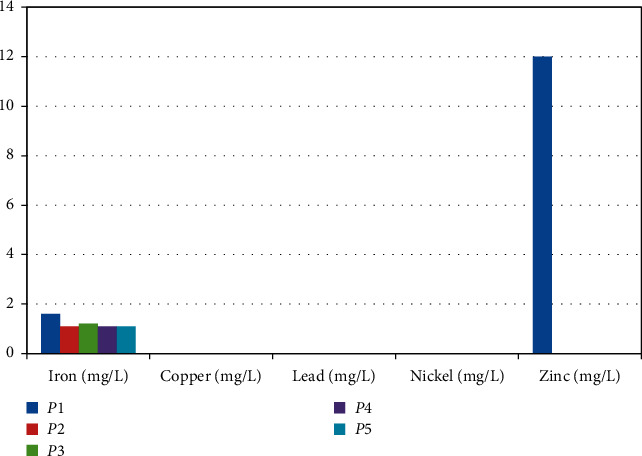
Graph of heavy metal concentration.

The samples of physicochemical parameters of food waste water were collected from renowned hotels of Bilaspur city are depicted in Tables 1 and 2. Figures 1 and 2 reveal the graph of physicochemical parameters and heavy metal concentration, respectively.

### 4.1. pH

pH (potential hydrogen) of a solution refers to its hydrogen ion activity and is expressed as the logarithm of the reciprocal of the hydrogen ion activity at a given temperature. pH of the samples ranged from 7.80 to 10.20 as presented in Table 1. Point *P*4 has the lowest value (7.80), while point *P*1 has the highest pH value of 10.20. The mean pH values recorded for all the sampling points are above the municipal authorities and WHO. Tolerance limit of pH varying from 6.0 to 9.0 for waste water can be discharged into the sewage line with an exception of point *P*1. As per the Bureau of Indian Standards [19], the permissible limit of pH in drinking water ranges from 6.5 to 8.5. The variation occurred in the pH values due to change in the values of CO_2_, carbonate, and bicarbonate in water. The lower values of pH may cause tuberculosis. Higher values may produce incrustation, sediment, deposition, and some difficulties in chlorination for disinfections of water [20]. In the present study, the pH values in all the collected water samples range from 9.0 to 10 except at point *P*1 which are all within the limit.

### 4.2. Temperature (°C)

Temperature is basically important for its effect on other properties of waste water. Temperature values for various samples are presented in Table 1, ranging from 31.15°C to 34.30°C. The highest value was found in site *P*1 followed by *P*3 and *P*2, while site *P*4 had the lowest temperature. Release of high-temperature waste water into water bodies may speed up some reactions in the water body. It will also reduce solubility of oxygen and amplified odor due to anaerobic reaction (less oxygen).

### 4.3. Turbidity

Turbidity values are found to be in the mean of 36.22 NTU for *P*1, 34.34 NTU for *P*2, 35.34 NTU for *P*3, 31.23 NTU for *P*4, and 32.33 NTU for *P*5.

### 4.4. Conductivity

Electrical conductivity is the measure of water capacity to convey electric current. Electrical conductivity of water is directly proportional to its dissolved mineral matter content [21]. The source of conductivity may be an abundance of dissolved salts due to addition of table salt in food materials, actual salt present in pure water, and other mineral discharges. The conductivity values are found to be 1335.21 *μ*s·cm^−3^ for *P*1, 1340.32 *μ*s·cm^−3^ for *P*2, 1460.32 *μ*s·cm^−3^ for *P*3, 1060.17 *μ*s·cm^−3^ for *P*4, and 1220.41 *μ*s·cm^−3^ for *P*5, refer Table 1. Conductivity of water which is a useful indicator of its salinity or total salt content is high in food waste water from the renowned hotels in Bilaspur. This result is not surprising since waste water from kitchen waste often contains high amounts of dissolved salts. The mean conductivity values for all the sampling points are higher than the municipal authorities and WHO guideline values of 1000 *μ*s·cm^−3^ for the discharge of waste water through hotels into sewages [22].

### 4.5. Total Dissolved Solids

A known amount of the water sample was filtered through a preweighed filter paper. The filter paper was then dried at 103°C–105°C. TSS was determined by using the following formula:

(1) $$\begin{array}{c} \text{TSS }\left(\text{mg}/\text{L}\right)=\frac{\left(\text{final wt}-\text{initial wt}\right)\times 1000}{\text{amount of the sample taken}}. \end{array}$$

The mean concentration of total dissolved solid (TDS) in the Bilaspur city is presented in Table 1. The concentration of TDS is recorded as 370.44 mg/L for *P*1, 255.23 mg/L for *P*2, 310.22 mg/L for *P*3, 390.20 mg/L for *P*4, and 360.20 mg/L for *P*5. These values obtained for TDS in all the sampling points were found below the WHO standard of 2000 mg/L for the discharge of waste water into surface water [23].

### 4.6. Total Suspended Solids

The total suspended solid (TSS) concentrations were found to be 300.22 mg/L for *P*1, 350.25 mg/L for *P*2, 300.27 mg/l for *P*3, 200.24 mg/L for *P*4, and 250.26 mg/L for *P*5 (Table 1). The Bureau of Indian Standards has specified a maximum limit of 100 mg/L for suspended solids in waste water, dischargeable into water courses. Results of the research work show that food waste water from the major hotels in Bilaspur can be classified as strong waste water and cannot be discharged into the stream [24]. Suspended solids may kill fish and other aquatic fauna by causing abrasive injuries by clogging the gills and respiratory passages, by blanketing the stream bottom, by destroying the spawning beds, and by screening out light necessary for the photosynthetic activity of aquatic plants. From the results of this study, the levels of TSS in the entire sample points exceeded the WHO guidelines of 50 mg/L for the protection of fisheries and aquatic life [25].

### 4.7. Total Alkalinity

Low alkalinity causes corrosion of pipes and increases the chance for releasing of many heavy metals. The permissible value of alkalinity as recommended by the Indian Standards is 200 mg/L for CaCo_3_. The amount of alkalinity concentration of the water sample collected in the study area ranged from 150 to 250 mg/L. Alkalinity and pH are the factors in determining the amenability of waste water to biological treatment.

### 4.8. Biological Oxygen Demand and Chemical Oxygen Demand

Biochemical oxygen demand is a measure of the quantity of oxygen consumed by microorganisms during the decomposition of organic matter. BOD and COD concentrations of waste water were measured as the two are of major concerns [5]. BOD_5_ was computed by subtracting DO after five days of incubation from DO measured on collection of samples at the point in mg/L. BOD_5_ = Do_*i*_ − Do_*f*_ or (Do_*i*_ − Do_*f*_) × volume of BOD bottle = *R*/Vol of the water sample taken. BOD concentration of waste water obtained for points *P*1 to *P*5 ranged between 100.15 and 165.25 mg/L, respectively. The concentrations of BOD and COD in all the sampling points are higher than the WHO values of 50 mg/L and 1000 mg/L for the discharge of waste water into the stream. High COD and BOD concentrations observed in waste water might be due to the use of chemicals. The results for elemental concentration in food waste water samples from renowned hotels in Bilaspur city for different sampling points are shown in the table. Chemical oxygen demand (COD) is the oxygen requirement of a sample for oxidation of organic and inorganic matter. The blank titration was carried out as mentioned above but using distilled water in place of the sample. The COD of the water sample was calculated from the following expression: COD (mg/L) = normality of ferrous ammonium sulphate × 8 × 1000 = *R*/mL sample taken for estimation. Waste water has an average COD concentration of 160.35 to 210.30 mg/L for points *P*1 to *P*5, refer Table 1.

### 4.9. Dissolved Oxygen

DO is a measure of the degree of pollution by organic matter and the destruction of organic substances, as well as the self-purification capacity of the water body. The maximum tolerance limit for fish is 5 mg/L, and below 2 mg/L leads to death [26]. Dissolved oxygen (DO) values obtained for points *P*1 and *P*2 varied between 6.25 mg/L and 8.40 mg/L, as observed in Table 1. The DO level at points *P*1 to *P*5 was above these levels.

### 4.10. Total Organic Carbon

Total organic carbon at point *P*1 was found to be 1.5%, which was the highest value amongst five points, and the lowest value recorded was 1.0% at points *P*2 and *P*3.

### 4.11. Sulphate, Nitrate, and Phosphate

The concentrations of sulphate, nitrate, and phosphate in all the sampling points varied between 80.45 mg/L and 110.35 mg/L for sulphate, 20.15 mg/L and 30.35 mg/L for nitrate, and 10.33 mg/L and 12.65 mg/L for phosphate, respectively, refer Table 1. High concentration of sulphate, nitrate, and phosphate was observed at point *P*1, while low concentrations varied at different points for sulphate (*P*2), nitrate (*P*2), and phosphate (*P*4). High amounts of sulphate impart bitter taste to water [27]. Sulphate as magnesium sulphate causes laxative effects to children particularly in hot weather or climates. Sulphate concentrations in the five sampling points ranged between 85.25 and 115.35 mg/L. Point *P*2 showed the lowest concentration, while the highest level of sulphate was observed at point *P*1. The levels of nitrate exceeded the WHO limits of 45 mg/L and the Indian guideline of 0.20 mg/L. Nitrate concentration was above the limit, while sulphate was below the WHO limit of 200 mg/L for the discharge of waste water into sewage. In addition to naturally occurring nitrates, it is also contributed to water sources by the application of fertilizers to lands [28]. Nitrate levels in the five sampling points fluctuate between 25.15 mg/L and 30.30 mg/L. The highest nitrate value was observed at site *P*1, while site *P*2 shows the least value. Nitrate, a compound of nitric acid, is the most highly oxidized form of nitrogen found in aquatic environment [29–31]. It is an essential nutrient for many photosynthetic autotrophs and in some instances, functions as a growth-limiting nutrient. It is used by algae and other aquatic plants to form plant protein which, in turn, can be used by animals to form animal protein. Nitrate is a major ingredient of farm fertilizers and is necessary for plant uptake and is essential for plant growth [2]. Nitrates are the indirect source of food for fish. This may increase the fish population. However, if algae grow too wildly, oxygen levels will be reduced and fish will die. The nitrate water quality guideline established by CCME for the protection of aquatic life is 13 mg/L. The levels of phosphate in all the sampling points are higher than the WHO limit of 5 mg/L. Polyphosphates are detrimental in that they interfere with coagulation, flocculation, and lime soda treatment of water. The levels of nitrate may give rise to methaemoglobinemia [32] in infants; also, the levels of nitrate reported in this study in addition to phosphate levels can cause eutrophication and may pose a problem for other uses. The highest concentration of phosphate was observed at site *P*1, while site *P*4 shows the least concentration. Phosphates are mostly from fertilizers, pesticides, industry, and cleaning compounds. Natural sources include phosphate-containing rocks and solid or liquid wastes. The element phosphorus is necessary for plant and animal growth. Nearly all fertilizers contain phosphates. Phosphates enhance the growth of plankton and water plants that serve as food for fish and aquatic life which results in increase of fish population that improves the quality of aquatic life. If excess phosphate is present, it may result in eutrophication. Many fish and aquatic organisms may not survive [33]. The huge agricultural activities coupled with the use of fertilizers, pesticides and other agrochemicals within the study area might have been responsible for the levels of phosphate in the water samples. In water, phosphorus is often biologically unavailable as it binds readily to particles. Soluble phosphorus which is available for uptake is called phosphate. Phosphates are not harmful to people or animals unless they are present in very high concentrations. The levels of phosphate in all the points exceeded the WHO maximum permissible limit of 5 mg/L [34].

### 4.12. Heavy Metals

Based on the assessment, it is drawn that the restaurant at point *P*1 is significantly discharging high levels of iron, which is due to corroded pipeline used for the supply of water. It is a well-known fact that iron is essential for the human body. Hence, it can be concluded that the low limit placed upon these metals in the standards has no health significance, and the limits are based on aesthetic and taste considerations [19]. The highest concentration of Fe 1.6 mg/L in the food waste water sample was detected at point *P*1, while the lowest value of 1.1 mg/L was observed at points *P*2, *P*4, and *P*5. Iron concentration was generally high in the entire sample analyzed. Although, iron is one of the essential elements in human nutrition, however, its presence at elevated concentration in aquatic ecosystems poses serious pollution and health problems [35]. Vomiting, cardiovascular collapse, and diarrhea have been reported due to deficiency of iron, while it may lead to failure of blood clotting. According to the WHO guideline value, maximum contaminant levels of 0.3 mg/L (water) for Fe are acceptable. Above 0.3 mg/L might lead to pollution of the aquatic environment. From the result of this study, the concentration of iron in water has exceeded the guideline limits indicating severe pollution. Copper, lead, and nickel are found below the detectable limit through analytic and spectrophotometric determination. The level of Zn in the samples of water collected from point *P*1 (site) was found richer than other sites, which shows that water is danger for aquatic life. Zn plays a biochemical role in the life processes of all aquatic plants and animals; therefore, it is essential in the aquatic environment in trace amounts. Zinc is used in a number of alloys including brass and bronze, batteries, fungicides, and pigments. Zinc is an essential growth element for plants and animals, but at elevated levels, it is toxic to some species of aquatic life [36]. In addition, Zn plays a vital role in a variety of enzyme systems which contribute to energy metabolism, transcription, and translation. Zinc is used in galvanizing steel and iron products. Zinc carbonates are used as pesticides [37]. Zinc was determined and obtained as 12 mg/L at point *P*1 which is slightly above the permissible limits for sustaining of aquatic life. Although zinc poses no known adverse physiological effects upon human beings, it poses adverse effects on aquatic organisms [32]. It is reported that concentrations of zinc in soft water ranging from 0.1 to 1.0 mg/L are lethal to fish. As per the BIS and other bodies, standard limit for drinking water ranges from 5 to 15 mg/L for human beings. Since zinc has a toxic effect towards protozoa and bacteria, the presence of even 0.1 mg/L zinc causes an appreciable fall in BOD [34].

## 5. Conclusions

Based on the results of this research, it was observed that the physicochemical levels of pH, turbidity, COD, DO, TOC, sulphate, nitrate, and phosphate of point *P*1 were recorded as highest values, where conductivity, total alkalinity, and BOD were recorded highest in point *P*3; rest of all parameter values at different points are recorded as lowest values. Overall, point *P*1 was discharging pollutants exceeding the limits of recommended guidelines of local bodies, WHO, ICMR, BIS, and CPCB standards, whereas point *P*4 values were found to be less polluting in the environment, and the values obtained were minimum. It was also observed that the levels of all the metals in the water samples exceeded the WHO, CPCB, ICMR, BIS, and local municipal authorities' limits. Based on the result of metal ion concentration analysis, the level of contaminants in the samples analyzed collected from the five food waste water-producing points was in the following order such as *P*1 shows 1.6 mg/L, *P*3 shows 1.2 mg/L, and other points *P*2, *P*4, and *P*5 show 1.1 mg/L. The metal ion concentration is found rich in case of *P*1 than other points. Similarly, other metal ion concentrations such as copper, lead, and nickel are also found beyond the detectable limit. The WQI was calculated by using the standards of the drinking water quality index, recommended by the World Health Organization (WHO), Bureau of Indian Standards (BIS), and Indian Council for Medical Research (ICMR). The weighted arithmetic index method [38] was used for the calculation of WQI of food waste water. Furthermore, quality rating or subindex (*qn*) is calculated using the following expression: *qn* = 100 [*V*_*n*_ − *V*_*io*_]/[*S*_*n*_ − *V*_*io*_] for further research work. The concentration of contaminants in all the effluents studied was mostly above the set limits by the WHO, ICMR, and CPCB in India. Due to the presence of high levels of toxic heavy metals, this waste water may not be good for irrigation in order to avoid accumulation of these metals in soils, and if the effluent is released into the environment without proper treatment, it may affect underground water and aquatic life. The conducted research revealed that waste water used for the irrigation and agricultural purposes of these nearby areas can be considered as remarkably polluted, and thus, it is not suitable for agricultural and aquatic bodies. It is concluded that the pollution is found due to lacking of regular cleaning of storage tanks or washing the plate along with residue/left over foods by customer in investigated restaurants. Plates with left foods are washed with water, which is released to earth, release the toxic pollutants and produce the pollution.

## Figures and Tables

**Table 1:** The highest value of different parameters recorded in five points is plotted in a graph.

Sr. no	Parameters determined	Sampling points				
		*P*1	*P*2	*P*3	*P*4	*P*5
1	pH	10.20	9.30	9.25	7. 80	9.65
2	Temp (°C)	34.30	31.30	33.30	31.15	30.40
3	Turbidity (NTU)	36.22	34.34	35.34	31.23	32.33
4	Conductivity (*μ*s·cm^−3^)	1335.21	1340.32	1460.32	1060.17	1220.41
5	TDS (mg/L)	370.44	255.23	310.22	390.20	360.20
6	TSS (mg/L)	310.25	210.30	260.30	360.30	310.30
7	Total alkalinity (mg/L)	200	220	250	165	150
8	BOD (mg/L)	160.15	150.25	165.25	120.45	100.15
9	COD (mg/l)	210.30	195.15	210.15	170.35	160.35
10	DO (mg/L)	8.40	6.45	7.87	6.25	6.30
11	TOC (%)	1.5	1.0	1.0	1.15	1.25
12	Sulphate (mg/L)	115.35	85.25	85.50	86.35	110.30
13	Nitrate (mg/L)	30.30	25.15	20.30	20.25	25.35
14	Phosphate (mg/L)	12.75	10.50	12.45	10.35	10.40

**Table 2:** Heavy metal concentration in water.

1	Iron (mg/L)	1.6	1.1	1.2	1.1	1.1
2	Copper (mg/L)	BDL	BDL	BDL	BDL	BDL
3	Lead (mg/L)	BDL	BDL	BDL	BDL	BDL
4	Nickel (mg/L)	BDL	BDL	BDL	BDL	BDL
5	Zinc (mg/L)	12	BDL	BDL	BDL	BDL

BDL: below detectable limit.

## Data Availability

The data used to support the findings of this study are available from the corresponding author upon request.
